# The Evaluation of Machine Learning Models Using Matrix‐Assisted Laser Desorption/Ionization Time‐of‐Flight Mass Spectrometry (MALDI–TOF–MS) Spectra for the Prediction of Antibiotic Resistance in *Klebsiella pneumoniae*

**DOI:** 10.1002/mbo3.70257

**Published:** 2026-03-02

**Authors:** Stephen Mark Edward Fordham

**Affiliations:** ^1^ Department of Life & Environmental Sciences Bournemouth University Poole UK

**Keywords:** *Klebsiella pneumoniae*, machine learning, MALDI–TOF–MS, rapid diagnostics

## Abstract

Antimicrobial resistance in *Klebsiella pneumoniae* poses a major clinical challenge, driving development in rapid, diagnostic strategies that extend beyond conventional susceptibility testing. Twenty‐three studies demonstrate that using matrix‐assisted laser desorption/ionization time‐of‐flight mass spectrometry (MALDI–TOF–MS) spectra to create machine learning (ML) models yields rapid and accurate predictions of antibiotic resistance in *K. pneumoniae*. Across these studies, most models focused on carbapenem resistance and achieved Area Under the Receiver Operating Characteristic Curve (AUROC) values consistently above 0.90, with ensemble algorithms, particularly Random Forest, XGBoost, and Light Gradient Boosting Machine, and deep learning models such as Convolutional Neural Networks attaining accuracies as high as 97% and even AUROCs reaching 0.99 or higher. Sample sizes ranged from 35 to over 15,000 isolates, reinforcing the robustness of these findings across diverse clinical settings. In addition to high discrimination performance, this evaluation reports that ML models developed using MALDI–TOF–MS spectra shorten diagnostic turnaround from days (48–96 h with conventional methods) to minutes or hours, using existing MALDI–TOF–MS equipment for economical implementation. However, ML diagnostic tools remain constrained by limited external validation, spectra preprocessing protocols, and variability between different MALDI–TOF–MS platforms. These limitations may restrict model generalizability and clinical translation, highlighting the need for standardized workflows and larger multicenter evaluations.

## Graphical abstract

Machine learning models developed from matrix‐assisted laser desorption/ionization time‐of‐flight mass spectrometry spectra can rapidly predict antimicrobial resistance in *Klebsiella pneumoniae*. By transforming routine spectral data into accurate resistance profiles within minutes, this approach could reduce diagnostic delays and support earlier targeted therapy, improving infection management and antimicrobial stewardship. However, current models are limited by external validation data sets, which may restrict model generalizability on unseen data, a problem compounded by different lab workflows used to obtain spectra.

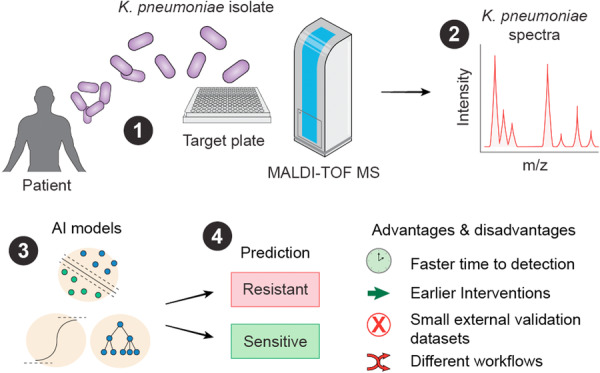

## 1 Introduction

Antibiotic resistance is a global health problem. Deaths attributable to antibiotic resistance are predicted to increase to 10 million each year by 2050 (O'Neill 2016). Antibiotic resistance imparts both an economic and healthcare burden. In 2018, the cost of managing antimicrobial‐resistant infections in England was estimated at approximately £180 million per year for the National Health Service (NHS) (Committee of Public Accounts 2025). In the United States, managing infections caused by six of the Centers for Disease Control and Prevention's highest‐priority antimicrobial resistance (AMR) threats is estimated to add over $4.6 billion in healthcare costs each year (CDC 2025). Antibiotic resistance is also associated with significantly elevated mortality and readmission risk (Poudel et al. 2023). Efforts to drive down the economic and healthcare impact of antibiotic resistance are desperately needed.

Traditionally, in the clinic, antibiotic resistance can be detected using widely approved molecular techniques and phenotypic assays. Molecular techniques are often narrow‐spectrum, with single gene targets, while phenotypic assays, such as culture‐based methods, require up to 48–72 h from sample collection until resistance reporting (Cordovana et al. 2018).

Matrix‐assisted laser desorption/ionization time‐of‐flight mass spectrometry (MALDI–TOF–MS) instruments are widely used across microbiology laboratories, including both clinical and research settings, for the rapid identification (ID) of bacterial species, fungi, and certain mycobacteria. The two main systems in use are the Bruker Daltonics MALDI Biotyper and the bioMérieux VITEK MS, both of which are Conformité Européenne – In Vitro Diagnostic (CE‐IVD) marked and routinely employed in NHS laboratories as frontline tools for microbial ID (Park et al. 2021). These systems have largely replaced traditional biochemical tests due to their speed, accuracy, and cost‐effectiveness, providing results within minutes once a pure colony is available (Clark et al. 2013; Osthoff et al. 2017). Their use is supported by United Kingdom Accreditation Service‐accredited standard operating procedures and aligns with guidelines issued by the UK Health Security Agency (UKHSA). Institutions such as UKHSA reference laboratories, University hospitals such as Oxford, Cambridge, Imperial College London, veterinary services, environmental monitoring labs, and food safety testing facilities all rely on MALDI–TOF–MS for microbial diagnostics (Singhal et al. 2015).

While this process is rapid, it does not provide information about antibiotic resistance. Empiric antibiotics are administered until detailed susceptibility results become available. Although empiric antibiotics can help treat the presumed pathogens, optimal treatment is only possible once a comprehensive resistance profile is obtained. Conventionally, MALDI–TOF–MS proteomics‐based bio typing relies on a small number of attributes derived from ribosomal proteins, including peak height and the area under the peak, to identify the particular microbial species via comparison between extensive and validated microbial library spectra. While this approach works well for the ID of microorganisms, there is a wealth of information contained within these spectra that remains unused. To fully exploit the information within the spectra, researchers have been developing machine learning (ML) models to predict antibiotic resistance based on MALDI–TOF–MS spectra. Resistance prediction can be obtained within 24 h of sample collection, 24–48 h earlier than traditional phenotypic reporting (Jian et al. 2024b).

ML methods are well‐suited for antibiotic resistance model prediction. ML models can find statistical dependencies in the data and can consider nonlinear and interactional effects between different features. Features derived from MALDI–TOF–MS spectra, combined with sensitivity‐labeled data for a particular antibiotic, can be used to create training and test data sets for predictive model development. The Sci‐Kit learn library in Python provides ML and hyperparameter tuning for optimal model development (https://scikit-learn.org/stable/).

MALDI–TOF–MS coupled with ML has been applied for the prediction of antibiotic resistance in *Klebsiella pneumoniae*. The integration of ML for resistance prediction using MALDI–TOF–MS proteomic peak data may be a useful addition to the routine clinical workflow. Improved time‐to‐resistance detection may support clinical decision‐making and contribute to antibiotic stewardship programs. Rapid detection of resistant infections may hasten effective antibiotic administration, thereby improving clinical outcomes in patients.

In a recent retrospective clinical case analysis, an ML model predicted the presence of carbapenem‐resistant *K. pneumoniae* (CRKP) in clinical samples from 34 patients. Confirmatory analysis later identified CRKP in 24 (70.6%) of the patients. On average, an interval of 1.4 days between the initial ML prediction and final culture report was observed (Yu et al. 2023). In addition, 19/24 (79.2%) of the patients with CRKP infections received inappropriate empirical antibiotics. Notably, the mortality rate was high, 42.1% (8/19), in patients who received inappropriate antibiotics. In contrast, a lower mortality rate, 28.6% (4/14), was observed in patients who received inappropriate antibiotics, but who subsequently underwent antibiotic regimen adjustment following the initial ML notification of CRKP status (Yu et al. 2023). The ML model provided critical time savings and changed the antibiotic treatment regimen, improving patients' outcomes. This is especially important, as it has been proposed that every hour delay in effective antibiotic administration reduces survival by 7.6% (Kumar et al. 2006).

Investigating ML models trained on MALDI–TOF–MS spectra for *K. pneumoniae* exploits data already generated in routine diagnostics to deliver rapid, actionable insights into AMR. According to national surveillance data submitted by 104 countries to the WHO (2025) Global Antimicrobial Resistance and Use Surveillance System (GLASS), 60.4% (95% CrI, 54.0–66.5) of *K. pneumoniae* bloodstream infections were resistant to the third‐generation cephalosporin ceftriaxone, while 46.4% (95% CrI, 40.9–52.1) showed resistance to the fourth‐generation cephalosporin cefepime (GLASS 2025). Imipenem resistance is also rising, with a median annual relative increase of 15.3% (95% CrI, 12.7–18.1) between 2018 and 2023 and an estimated global prevalence of 16.7% (95% CrI, 13.8–20.1) in 2023, based on data from 64 countries (GLASS 2025). These alarming resistance levels and upward trends highlight *K. pneumoniae* as a global priority pathogen, justifying its focus. Transforming MALDI–TOF–MS from a species ID tool into a predictive platform for resistance profiling could transform clinical microbiology, enabling earlier, targeted therapy, improving patient outcomes, and strengthening antimicrobial stewardship worldwide.

## 2 Method

A structured search was conducted to identify studies evaluating the application of ML models to MALDI–TOF–MS spectra for the prediction of antibiotic resistance in *K. pneumoniae*. The search strategy used the string terms: “*Klebsiella pneumoniae*” AND “machine learning” AND “Matrix‐Assisted Laser Desorption/Ionization Time‐of‐Flight Mass Spectrometry” AND “antibiotic resistance.” The search covered the period from January 2017 to October 2025 and was performed across three databases: PubMed (NCBI), Scopus, and Google Scholar. Only open‐access, peer‐reviewed primary research articles were included. Gray literature such as preprints, theses, and conference posters or abstracts, as well as systematic reviews, was excluded. All search results were deduplicated prior to screening (Table S1).

From each article, data were collected on sample size (number of *K. pneumoniae* isolates), ML algorithms employed, resistance targets, reported Area Under the Receiver Operating Characteristic Curve (AUROC) and accuracy ranges, best‐performing model, and whether external validation was performed independently of the internal train‐test split test data set. Where available, the size of the external validation data set, the combined MALDI–TOF–MS and ML time‐to‐resistance reporting, and the traditional phenotypic or molecular method used for comparison and its corresponding turnaround time were also recorded. Limitations of each ML model were extracted as reported by the authors. Extracted information was subsequently determined to evaluate methodological approaches, model performance, and the translational potential of ML‐based MALDI–TOF–MS workflows for rapid resistance prediction in *K. pneumoniae*.

## 3 Results

### 3.1 Characteristics of ML‐Based MALDI–TOF–MS Studies for the Prediction of Antibiotic Resistance in *K. pneumoniae*

A total of 23 studies met the inclusion criteria and were included for data extraction. This evaluation included these 23 studies that investigated the application of MALDI–TOF–MS spectra with ML models for the prediction of antibiotic resistance in *K. pneumoniae*. Most studies adopted a retrospective design, with only four employing prospective data collection. The majority were conducted at single medical centers or within small networks of affiliated hospitals, which may limit the generalizability of their findings (Table S1).

**Figure 1:**
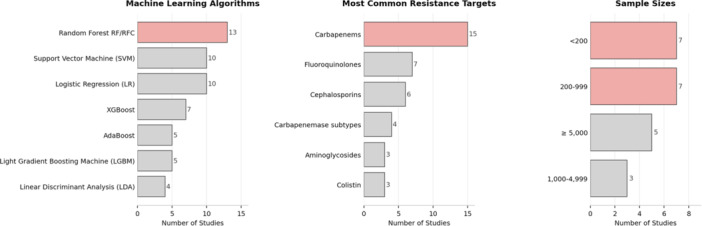
A quantitative summary of the characteristics of ML classifiers for the prediction of antibiotic resistance in *Klebsiella pneumoniae*. The most common ML classifiers include the RF model (13 studies), alongside SVC/LR (10 studies each). These ML classifiers have been developed to determine carbapenem resistance and/or their subtypes, and four other antibiotic resistance classes. A range of bacterial isolates is used to build these models; 35–15,782 isolates were included. Red colored horizontal bars refer to the highest counts obtained. LR, logistic regression; ML, machine learning; RF, Random Forest; RFC, RF classifier; SVC, support vector classifier.

A quantitative evaluation of the studies summarized in Table 1 highlights several consistent methodological and biological trends in the application of MALDI–TOF–MS data for predicting AMR in *K. pneumoniae*. Among the evaluated ML approaches, ensemble and kernel‐based algorithms predominated (Table 1). Random Forest (RF) models were the most frequently implemented (13 studies), followed by support vector machines (SVM, 10) and logistic regression (LR, 10), XGBoost (7), AdaBoost (5), and Light Gradient Boosting Machines (LGBMs, 5), and linear discriminant analysis (LDA, 4) (Figure 1). This distribution reflects a clear preference for models capable of handling nonlinear feature relationships and high‐dimensional spectral inputs.

**Table 1:** Machine learning (ML) algorithms developed to detect resistance targets in *Klebsiella pneumoniae*.

Reference	Study focus	*K. pneumoniae* isolates	ML algorithms employed	Resistance targets	Geographic origin
De Carolis et al. (2017)	Rapid diagnostic workflow for cefotaxime‐resistant *K. pneumoniae* from blood cultures	35	Custom classification algorithm	Cefotaxime	Italy
Giordano and Barnini (2018)	Rapid detection of colistin‐resistant *K. pneumoniae*	139	Genetic algorithm, supervised neural network, quick classifier	Colistin	Italy
Cordovana et al. (2018)	Full MALDI‐based approach to detect plasmid‐encoded KPC‐producing *K. pneumoniae*	6209	Algorithm integrated into MALDI Biotyper	Carbapenems (KPC detection)	Italy and Germany
Huang et al. (2020)	Detection of carbapenem‐resistant *K. pneumoniae* using supervised ML	95	RF, SVM, nearest neighbors, naïve Bayes, logistic regression (LR)	Carbapenems (imipenem, meropenem)	Taiwan
Gato et al. (2021)	Improved pipeline for identification (ID) of carbapenemase‐producing *K. pneumoniae*	162	PLS‐DA, RF	Carbapenems (KPC, NDM, Oxacillinase‐48 (OXA‐48))	Spain
Wang et al. (2022)	Large‐scale rapid detection of ciprofloxacin resistance	15,782	LR, SVM, RF, XGBoost	Ciprofloxacin	Taiwan
Weis et al. (2022)^a^	Species‐specific ML classification of antimicrobial resistance in *K. pneumoniae* from MALDI–TOF–MS spectra	Ceftriaxone: ~4000–4500/> 500 test isolates; other antibiotics: not reported^a^	Light Gradient Boosting Machine (LGBM), MLP, LR	Ceftriaxone (ESBL proxy), cefepime, ciprofloxacin, meropenem, piperacillin–tazobactam, tobramycin	Switzerland
Gato et al. (2023)	Direct detection of carbapenemase‐producing *K. pneumoniae*	715	RF, PLS‐DA, SVM, *k*‐NN	Carbapenems (meropenem, ertapenem), OXA‐48, KPC	Spain
Iskender et al. (2023)	Rapid determination of colistin resistance	260	LDA, SVM, ensemble	Colistin	Turkey
Yu et al. (2023)	Rapid prediction of carbapenem‐resistant *K. pneumoniae* from blood cultures	126	LGBM	Carbapenems	Taiwan
Zeng et al. (2023)	Prediction of imipenem resistance	174	LASSO, LR, SVM, neural network	Imipenem	China
Zhang et al. (2023)	Rapid ID of carbapenem‐resistant *K. pneumoniae* using artificial neural network (ANN)	2683	ANN	Carbapenems (ertapenem, imipenem, doripenem, meropenem)	Taiwan
De Waele et al. (2025)^a^	Multidrug MALDI–TOF–MS–based antimicrobial recommendation for *K. pneumoniae* using deep learning	Not reported per antibiotic; species‐specific recommender trained on *K. pneumoniae* subset of DRIAMS‐A (counts reported as spectrum–drug labels, not isolates)^a^	Dual‐branch neural network (recommender system)	Multidrug AMR profiles (56 antibiotics)	Switzerland
Jian et al. (2024a)	AI‐clinical decision support system for predicting resistance to levofloxacin and ciprofloxacin	11,996	LR, LDA, RF, Gradient Boosting, AdaBoost, XGBoost, LGBM	Levofloxacin, ciprofloxacin	Taiwan
Lin et al. (2024)	AI‐CDSS for ceftazidime‐avibactam resistance detection	675	RF, GBC, AdaBoost, XGBoost, LGBM, LR, LDA	Ceftazidime‐avibactam	Taiwan
Jian et al. (2024b)	AI‐CDSS for carbapenem‐ and colistin‐resistant strains	4307	RF, LR, LDA, GBC, AdaBoost, XGBoost, LGBM, SVM	Carbapenems (doripenem, imipenem), colistin	Taiwan
Lopez‐Cortez et al. (2024)	AMR prediction via deep neural networks and transfer learning	2800	Convolutional neural network (CNN) (MSDeepAMR)	Ciprofloxacin, ceftriaxone, cefepime, meropenem, tobramycin	Data derived from DRIAMS B–C–D data sets
Xu (2024)	Rapid detection of carbapenem‐resistant *K. pneumoniae*	240	RF, SVM, LR, XGBoost	Carbapenems (ertapenem, imipenem, meropenem)	China
Xu and Gao (2025)	Prediction of susceptibility to nine antibiotics	484	RF, XGBoost, AdaBoost, LR, MLP, SVM	Piperacillin/tazobactam, ceftazidime, ceftriaxone, cefotetan, aztreonam, imipenem, amikacin, levofloxacin, Co‐trimoxazole	China
Lin et al. (2025)	AI‐CDSS for predicting resistance to 12 antibiotics	12,967	RF, LGBM	Amikacin, gentamicin, β‐lactams, carbapenems, fluoroquinolones	Taiwan
Ye et al. (2025)	Rapid ID of carbapenemase subtypes	205	CNN, RF, SVM, AdaBoost	Carbapenems (KPC, NDM, and OXA‐48)	China
López‐Cortés et al. (2025)	Integrating ML with MALDI‐TOF for resistance detection	187	SVM, RF, LR, CatBoost	Ciprofloxacin	Chile
Xu et al. (2025)	Classification of carbapenem‐resistant *K. pneumoniae* from blood cultures	444	Decision tree, RF, Gradient Boosting Machine, XGBoost, extremely randomized trees	Carbapenems (ertapenem, imipenem, meropenem)	China

Abbreviations: AI, artificial intelligence; AMR, antimicrobial resistance; CDSS, clinical decision‐support systems; DRIAMS, Database of Resistance in Antimicrobials using MALDI‐TOF MS Spectrometry; ESBL, extended spectrum β‐lactamase; GBC, gradient boosting classifier; *k*‐NN, *k*‐nearest neighbors; KPC, Klebsiella pneumoniae carbapenemase; LASSO, least absolute shrinkage and selection operator; LDA, linear discriminant analysis; MALDI, matrix‐assisted laser desorption/ionization; MLP, multilayer perceptron; MS, mass spectrometry; NDM, New Delhi metallo‐β‐lactamase; PLS‐DA, partial least squares discriminant analysis; RF, Random Forest; SVM, support vector machine; TOF, time‐of‐flight.

a Weis et al. (2022) explicitly reported *K. pneumoniae* isolate counts for a single antibiotic (ceftriaxone; > 500 test isolates), while other antibiotic pairings lacked reported sample sizes. De Waele et al. (2025) did not report per‐antibiotic isolate counts, as resistance prediction was framed as a multidrug recommender task using spectrum–drug pairs rather than binary antibiotic‐specific classifiers.

The resistance phenotypes most commonly targeted centered on clinically significant mechanisms of treatment failure. Carbapenem resistance was the predominant focus (15 studies), underscoring its global relevance in *K. pneumoniae* epidemiology. Fluoroquinolone resistance (ciprofloxacin or levofloxacin) was investigated in seven studies, while cephalosporin resistance featured in six. Four studies examined specific carbapenemase families or subtypes, and smaller numbers addressed aminoglycoside (*n* = 3) and colistin (*n* = 3) resistance. Collectively, these efforts highlight the growing potential of MALDI–TOF–MS‐based ML approaches for addressing the complex diagnostic challenges posed by multidrug‐resistant *K. pneumoniae*.

Sample sizes, where specified, varied markedly across studies, reflecting differences in strain availability and study design. Seven investigations analyzed fewer than 200 isolates, and another seven included 200–999 isolates (Figure 1). Three studies incorporated 1000–4999 isolates, while five leveraged large‐scale data sets comprising ≥ 5000 isolates. Overall, sample sizes ranged from 35 to 15,782 isolates, with a median of 675, indicating that while large data sets are used to build predictive ML models, most MALDI–TOF‐based AMR prediction studies remain moderately scaled. The geographic distribution showed concentration in East Asian institutions (Taiwan and China) with additional contributions from European countries (Spain, Italy, Germany, Switzerland, and Turkey) and South America (Chile).

### 3.2 ML Algorithm Performance

Across the studies, the performance of ML algorithms for predicting antibiotic resistance was consistently high, with many models achieving an AUROC greater than 0.90 (Table 2). A clear trend emerged from the findings: ensemble and deep learning models frequently outperformed simpler, linear algorithms (Table 2). Tree‐based ensemble models, including RF, XGBoost, and Light Gradient Boosting Machine (LGBM), were often identified as the best‐performing algorithms. Huang et al. (2020) found that RF outperformed four other algorithms, achieving 97% accuracy in differentiating carbapenem‐resistant isolates, while Jian et al. (2024a) and Xu (2024) also identified RF as the top performer, with AUROCs of 0.96–0.98 and 0.99, respectively. Other gradient boosting models demonstrated strong results. Lin et al. (2025) highlighted LGBM's robust performance (AUROC 0.91–0.95), and Xu and Gao (2025) noted that XGBoost and AdaBoost achieved AUROCs of 0.80 or higher for all nine antibiotics tested.

**Table 2:** Machine learning model performance metrics and resistance targets in *Klebsiella pneumoniae*.

Reference	Algorithm(s) evaluated	Antibiotic target	Reported AUROC range	Reported accuracy range	Noted best‐performing model
De Carolis et al. (2017)	Custom classification algorithm (based on mass peaks)	Cefotaxime	Not stated	Agreement 92.5% with genotype	Direct MS β‐lactamase assay
Giordano and Barnini (2018)	Genetic algorithm, supervised neural network, quick classifier	Colistin	0.865	91.3%–99.8% (recognition)	Model based on two selected mass peaks
Cordovana et al. (2018)	Integrated peak detection algorithm (MALDI Biotyper)	Carbapenems (via KPC detection)	N/A	Sensitivity 85.1%, specificity 100%	Automated KPC‐peak detection
Huang et al. (2020)	Random forest (RF), SVM, nearest neighbors, naïve Bayes, logistic regression (LR)	Carbapenems (imipenem, meropenem)	Not stated	97% (RF)	RF
Gato et al. (2021)	PLS‐DA, RF	Carbapenems (meropenem, ertapenem); carbapenemase types (KPC, NDM, OXA‐48)	Not stated	100% (for CP‐*K. pneumoniae*)	RF‐M LINEAR method
Wang et al. (2022)	LR, SVM, RF, XGBoost	Ciprofloxacin	0.85–0.89	82%–83%	SVM and XGBoost
Weis et al. (2022)	LR, LightGBM, MLP	Ceftriaxone, cefepime, ciprofloxacin, meropenem, piperacillin–tazobactam, tobramycin	0.55–0.76	Not stated	MLP (ceftriaxone, cefepime), LightGBM (ciprofloxacin)
Iskender et al. (2023)	LDA, SVM, Ensemble	Colistin	0.94–0.99	81.6% (LDA test set)	LDA
Zeng et al. (2023)	LASSO, LR, SVM, neural network	Imipenem	0.97 (training set)	Not stated	LASSO algorithm
Gato et al. (2023)	RF, PLS‐DA, SVM, *k*‐nearest neighbor	Carbapenems (meropenem, ertapenem); carbapenemase types (OXA‐48, KPC)	1.00	95.24%–97.83%	RF
Yu et al. (2023)	Light Gradient Boosting Machine (LGBM)	Carbapenems	0.828	76.6%	LGBM (sole model tested)
Zhang et al. (2023)	Artificial neural network (ANN)	Carbapenems (ertapenem, imipenem, doripenem, meropenem)	0.91	84%	ANN
Lin et al. (2024)	RF classifier, gradient boosting classifier, AdaBoost, XGBoost, LGBM, LR, LDA	Ceftazidime‐avibactam	0.80–0.95 (test)	77%–90%	LGBM
Jian et al. (2024b)	RF classifier, LR, LDA, gradient boosting classifier, AdaBoost, XGBoost, LGBM, SVM	Carbapenems (doripenem, imipenem), colistin	0.95–0.98	87%–93%	RF
Jian et al. (2024a)	LR, LDA, RF, gradient boosting classifier, AdaBoost, XGBoost, LGBM	Levofloxacin, ciprofloxacin	0.68–0.95	64%–90%	RF
Lopez‐Cortez et al. (2024)	CNN (MSDeepAMR)	Ciprofloxacin, ceftriaxone, cefepime, meropenem, tobramycin	0.82–0.83	Not stated	Models for ceftriaxone and oxacillin resistance
Xu (2024)	RF, SVM, LR, XGBoost	Carbapenems (ertapenem, imipenem, meropenem)	0.96–0.99	90%–94% (test)	RF
Xu and Gao (2025)	RF, XGBoost, AdaBoost, LR, Multilayer Perceptron, SVM	Multiple antibiotics (piperacillin/tazobactam, ceftazidime, ceftriaxone, cefotetan, aztreonam, imipenem, amikacin, levofloxacin, Co‐trimoxazole)	0.63–0.99	Not stated	XGBoost
Ye et al. (2025)	CNN, RF, SVM, AdaBoost	Carbapenems; carbapenemase subtypes (KPC, NDM, OXA‐48)	0.99 (CNN)	96.1% (CNN)	CNN model
Lin et al. (2025)	RF classifier, LGBM	Multiple antibiotics (12 antibiotics, including amikacin, gentamicin, piperacillin/tazobactam, ceftazidime, ceftriaxone, cefoperazone/sulbactam, flomoxef, cefepime, imipenem, doripenem, ciprofloxacin, levofloxacin)	0.91–0.95	> 80%	LGBM
López‐Cortés et al. (2025)	SVM, RF, LR, CatBoost	Ciprofloxacin	0.60–0.73	57%–62% (balanced accuracy)	CatBoost

Abbreviations: AUROC, Area Under the Receiver Operating Characteristic Curve; CNN, convolutional neural network; CP, carbapenemase‐producing; KPC, Klebsiella pneumoniae carbapenemase; LASSO, least absolute shrinkage and selection operator; LDA, linear discriminant analysis; MALDI, matrix‐assisted laser desorption/ionization; MLP, multilayer perceptron; MLP, multilayer perceptron; MS, mass spectrometry; NDM, New Delhi metallo‐β‐lactamase; PLS‐DA, partial least squares discriminant analysis; RF‐M, rational function model; SVM, support vector machine.

Deep learning approaches, specifically Convolutional Neural Networks (CNNs), also showed excellent results. Ye et al. (2025) reported that their CNN model for identifying carbapenemase subtypes achieved an overall accuracy of 96.1% and an AUROC of 0.99, outperforming traditional ML models like RF and SVM. López‐Cortés et al. (2024) also reported strong performance with their deep learning model, with AUROC values from 0.82 to 0.83 for various antibiotics. The included studies show that combining MALDI–TOF–MS with ML can detect a wide spectrum of antibiotic resistance phenotypes in *K. pneumoniae*. Carbapenem resistance was the most extensively studied and successfully predicted category; numerous studies developed high‐performing models for identifying CRKP isolates, with AUROCs consistently above 0.90 (Table 2).

Beyond binary classification of resistance, several studies achieved a more granular level of detection. Ye et al. (2025) used a CNN to distinguish between KPC, NDM, and OXA‐48‐producing isolates with high accuracy, while Gato et al. (2023) also successfully predicted OXA‐48 and KPC carriage. A single mass peak associated with KPC‐producing strains, achieving 100% specificity, has also been developed (Cordovana et al. 2018).

ML predictive models using MALDI–TOF–MS spectra have also been successfully applied to other critical resistance profiles. Resistance to colistin was effectively predicted in three studies. Resistance to fluoroquinolones (ciprofloxacin and levofloxacin) was the focus of large‐scale studies that developed models with AUROCs up to 0.95. The approach has also been extended to newer agents, with Lin et al. (2024) developing a model to detect ceftazidime‐avibactam resistance with an AUROC of 0.95.

However, the predictive performance of these models varied depending on the antibiotic. While high accuracy was achieved for several drugs, levofloxacin proved the most challenging, with models showing the lowest classification ability (Xu and Gao 2025). The authors suggest this is because levofloxacin resistance arises from a variety of mechanisms, many of which do not produce distinct changes in the bacterial proteome detectable by MALDI–TOF–MS. The effectiveness of ML predictive models using MALDI–TOF–MS spectra relies on whether the resistance mechanism leads to measurable alterations in the bacterial protein profile.

The relationship between data set size and predictive performance in MALDI–TOF–MS‐based ML models for *K. pneumoniae* antibiotic resistance prediction is not strictly linear, with studies ranging from 35 to 15,782 isolates achieving AUROC values from 0.73 to 1.00 (Tables 1 and 2). Explicit size‐performance analyses suggest minimum thresholds of 2500 samples for high predictive performance and over 3000 samples for robust AUROC/AUPRC values (Weis et al. 2022; López‐Cortés et al. 2024). Smaller studies occasionally achieved comparable metrics; AUROC of 0.99 with only 240 isolates (Xu 2024) and 205 isolates (Ye et al. 2025), respectively. Conversely, one of the largest studies (15,782 isolates) reported a reduced AUROC score of 0.89, suggesting diminishing returns and indicating that data set quality, class balance, and resistance mechanism homogeneity may matter more than absolute size beyond minimum thresholds (Wang et al. 2022). Overfitting effects may also be pronounced with small training data sets.

Studies with temporal validation splits (training on earlier samples, validating on later samples) reported lower but potentially more realistic performance estimates. Lin et al. (2025) used January–September data for training and October‐December for validation, while Jian et al. (2024a) employed similar temporal approaches. These designs better reflect real‐world deployment scenarios where models must predict on future, potentially novel isolates. However, most size‐performance inferences derive from cross‐study comparisons confounded by differences in resistance prevalence, ML algorithms, preprocessing approaches, and validation strategies. The field would benefit from controlled studies holding these factors constant while systematically varying training set size.

### 3.3 Isolate Culture

**Figure 2:**
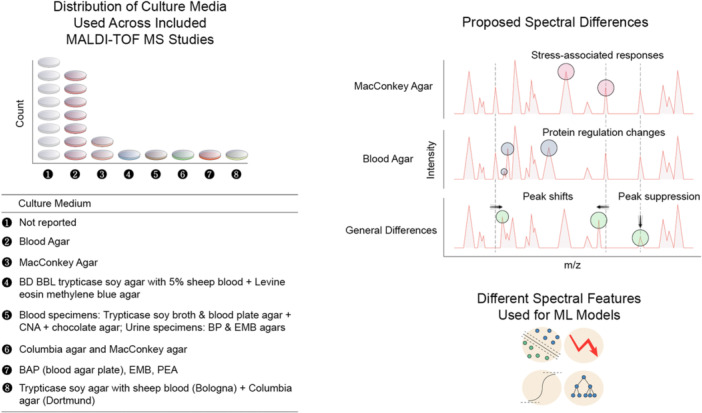
Agar medium used prior to spectral acquisition in *Klebsiella pneumoniae* for ML models. (Left) The predominant culture medium across studies was blood agar. Eight studies did not report the culture media used, representing a significant gap in methodological reporting. (Right) When classification relies on pattern‐matching across thousands of spectral features, even subtle changes in protein expression due to media composition may accumulate to affect prediction accuracy; small m/z peak shifts and unique peak and/or suppression may occur. The De Waele et al. (2025) study developed a MALDI–TOF–MS‐based antimicrobial recommender system and used the same DRIAMS core data set for the development of ML predictive models as Weis et al. (2022). This study was therefore not included in the “Not reported” category to eliminate duplication. BP, Baird–Parker; CNA, circulating nucleic acids; DRIAMS, Database of Resistance in Antimicrobials using MALDI‐TOF MS Spectrometry; EMB, eosin methylene blue; MALDI, matrix‐assisted laser desorption/ionization; ML, machine learning; MS, mass spectrometry; PEA, presumptive enteric agar; TOF, time‐of‐flight.

Bacterial growth conditions directly influence protein expression profiles. In turn, this alters the MALDI–TOF–MS spectra used for ML‐based AMR prediction. In the studies evaluated, there was considerable variability and frequent underreporting in the culture media used prior to spectral acquisition (Figure 2). When combining Columbia blood agar and nonspecific blood agar into a single category, solely blood‐based media accounted for seven studies, making this the most frequently used medium overall (Figure 2). MacConkey agar was employed in two studies, while more complex or multimedia workflows, including combinations of blood agar plates, eosin methylene blue agar, presumptive enteric agar, or laboratory‐specific formulations, appeared in five studies. However, the most notable finding was that eight studies did not report the culture medium, making “Not reported” the largest individual category (Figure 2 and Table S1). This variability and lack of reporting represent an important methodological gap, given the influence of media composition on MALDI–TOF–MS spectral output.

Direct evidence addressing the influence of agar media composition on ML model accuracy for predicting antibiotic resistance in *K. pneumoniae* was only included in 2 of 23 studies (8.7%). Gato et al. (2023) conducted the most comprehensive media comparison, systematically evaluating blood agar, chocolate agar, and MacConkey agar. The study found that culture medium was determinant in carbapenemase‐producing *K. pneumoniae* (CPK) prediction, with poor classification observed when isolates were cultured on chocolate agar or MacConkey agar compared with blood agar. The RF algorithm achieved 95.24% accuracy for OXA‐48 or KPC carriage prediction when isolates were cultured on blood agar (the same medium used to build the model). The AUROC and AUPRC both reached 1.00 for CPK prediction under standardized conditions (Gato et al. 2023).

Separately, another study compared Sheep Blood Agar, CHR‐KPC Agar, and Mueller‐Hinton‐Agar for automated peak detection of KPC‐producing strains. In contrast to Gato et al. (2023), this study observed no performance differences between these media types. The automated peak detection achieved 85.1% sensitivity and 100% specificity regardless of media type (Cordovana et al. 2018). It should be noted that this study focused on the detection of a specific KPC‐related peak at 11,109 m/z rather than comprehensive ML‐based resistance prediction.

### 3.4 Intensity Normalization Methods

Intensity normalization is critical in MALDI–TOF–MS‐based AMR modeling. During a preprocessing workflow, intensity normalization standardizes spectral signal scales across runs and samples, thereby reducing nonbiological intensity variation that can otherwise distort peak features and lead ML models to learn instrument‐ or preparation‐driven artefacts rather than true resistance‐associated signatures.

A comprehensive assessment of intensity normalization approaches revealed substantial heterogeneity across studies, with several studies failing to report normalization methods entirely. The evaluation revealed that only 14 of 23 studies (61%) explicitly reported their intensity normalization approach (Table S1). Among those reporting, four distinct categories of normalization methods emerged.

Four studies employed Total Ion Current (TIC) normalization, where each intensity value is divided by the sum of all intensities in the spectrum. Gato et al. (2021) demonstrated that TIC normalization reduced total interlaboratory imprecision by 40%, with the coefficient of variation decreasing from 26.0 for raw spectra to 15.6 for normalized spectra.

The square‐root transformation was applied in four studies, while the logarithmic transformation was consistently employed across four related studies (Table S1). *Z* score transformation using StandardScaler was additionally applied in one study (Zhang et al. 2023; Table S1). One study normalized spectrum densities using the highest intensity value (Iskender et al. 2023). Nine studies (39%), however, did not explicitly report their normalization methodology, representing a significant gap in methodological transparency.

Most studies (20 of 23) utilized a mass range of approximately 2000–20,000 Da, which aligns with the typical protein/peptide detection range for bacterial ID. Exceptions included one study using a narrower 100–1000 Da range for antibiotic hydrolysis product detection (De Carolis et al. 2017) and two studies restricting analysis to 2000–12,000 Da (Iskender et al. 2023; Xu and Gao 2025).

### 3.5 Isolate Source and External Validation

Only three studies (13%) validated their models using independent external data sets. López‐Cortés et al. (2024) validated models on external data sets from different clinical laboratories within the Database of Resistance in Antimicrobials using MALDI‐TOF MS Spectrometry (DRIAMS) database. De Waele et al. (2025) employed data from multiple hospitals for transfer learning experiments. Weis et al. (2022) additionally performed external validation using the DRIAMS data set. The remaining 20 studies relied exclusively on internal validation methods such as cross‐validation and train/test splits from the same data set.

External validation performance, where reported, was notably lower than typical internal validation results. López‐Cortés et al. (2024) achieved an AUROC of only 0.594 on the DRIAMS‐C external data set for *K. pneumoniae*‐Ceftriaxone prediction, compared with an internal validation AUROC value of 0.82. Several studies explicitly acknowledged the absence of external validation as a limitation affecting real‐world diagnostic accuracy and generalizability, with authors noting the need for multicenter studies to validate findings.

The isolates used to develop these models were sourced from a modest number of clinical sites, with the number of hospitals contributing data ranging from 1 to 15 (Table S1). In all, 12/23 (52.17%) studies derived isolates from a single hospital/institution, while a few incorporated multicentre collections. This limited sampling breadth may restrict the representativeness of the models when applied to broader or geographically distinct *K. pneumoniae* populations. The predominant reliance on single‐institution data and internal validation methods represents a significant gap in establishing the clinical readiness of these predictive models.

**Figure 3:**
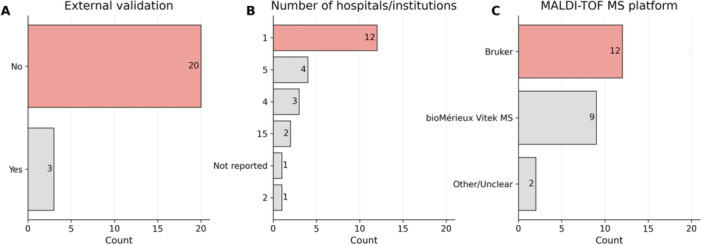
Platform, source, and external validation of isolates used to develop ML models for antibiotic prediction in *Klebsiella pneumoniae*. (A) External validation was performed for three studies. De Waele et al. (2025) used data from multiple hospitals (DRIAMS‐B, DRIAMS‐C, and DRIAMS‐D) for transfer learning experiments and external validation. The main training data came from DRIAMS‐A (University Hospital Basel), with external validation using 1000 randomly drawn spectra from each of the other hospitals. Weis et al. (2022) and López‐Cortés et al. (2024) validated models using the DRIAMS database, which includes external data sets from DRIAMS‐B, DRIAMS‐C, and DRIAMS‐D, collected from different clinical laboratories using the same mass spectrometry system. The external data sets were from different laboratories than the training data (DRIAMS‐A). (B) Most models are developed using data from a single hospital. (C) Bruker and VITEK bioMérieux MALDI–TOF–MS platforms are primarily used to acquire spectra for model development. DRIAMS, Database of Resistance in Antimicrobials using MALDI‐TOF MS Spectrometry; MALDI, matrix‐assisted laser desorption/ionization; ML, machine learning; MS, mass spectrometry; TOF, time‐of‐flight.

In terms of MALDI–TOF–MS instrumentation, the Bruker Microflex LT/SH (Bruker Daltonics GmbH) was the most frequently employed platform, used in 12 of the 23 studies (52%). The VITEK MS IVD system (bioMérieux, Lyon, France) was used in nine studies (39.1%), while one study (4.3%) utilized a MALDI–TOF–MS spectrometer manufactured by Chongqing ZhongyuanHuiji Co (Figure 3B). The predominance of Bruker and VITEK systems reflects their widespread adoption in clinical diagnostics and their role in ML workflows investigating AMR in *K. pneumoniae*.

### 3.6 Improved Time to Detection of ML Classifiers

Across the evaluated studies, integration of ML models with MALDI–TOF–MS workflows produced substantial reductions in the time required to determine antibiotic‐resistant phenotypes in *K. pneumoniae*. Conventional phenotypic assays, such as automated broth microdilution, disk diffusion, or VITEK‐based antibiotic susceptibility testing (AST) typically required between 1 and 4 days to provide sensitivity status, after a specimen has been collected, cultured, and subcultured for isolation prior to species ID via MALDI–TOF–MS. Crucially, the ML predictive models do not eliminate culture time, rather, they collapse the AST phase; ML‐based MALDI–TOF–MS predictions can be made as soon as colonies are available for species ID. Importantly, this step already exists in routine diagnostics.

Eight investigations reported turnaround times of 1 h or less, with several models achieving results in as little as 20 or 30 min, while one study identified CPK strains in real time using an RF model (Gato et al. 2023; Table 3). Two additional studies reported detection within 2 h, while three described approximately 3‐day workflows, typically representing AI‐driven clinical decision‐support systems (CDSS) integrated into routine hospital pipelines. For seven further studies, the time to result was qualitatively described as “rapid” but lacked quantitative specification. These results can be summarized in Table 3.

**Table 3:** Time‐to‐detection improvements in MALDI–TOF–MS + ML models for antibiotic resistance detection in *Klebsiella pneumoniae*.

Study	ML + MALDI‐TOF time to result	Traditional method and time	Time saved versus listed traditional methods	Comparator method	Antibiotic target
De Carolis et al. (2017)	< 2 h	VITEK‐2/E‐test, 24–48 h longer	24–48 h	Automated AST/E‐test	Cefotaxime
Giordano and Barnini (2018)	90 min	Broth microdilution, 20 h	~18.5 h	Broth microdilution	Colistin
Cordovana et al. (2018)	10–90 min	Up to 24 h	Up to 23 h	Commercial methods	Carbapenems (KPC detection)
Huang et al. (2020)	Not stated	VITEK‐2 (“time‐consuming”)	Not stated	Automated MIC	Carbapenems (imipenem, meropenem)
Gato et al. (2021)	Real‐time	EUCAST (not specified)	Not stated	Broth/disk	Carbapenems (KPC, NDM, OXA‐48)
Wang et al. (2022)	< 2 h	Disk, 48 h	~46 h	Disk diffusion	Ciprofloxacin
Yu et al. (2023)	1 h	36–72 h	35–71 h	Conventional ID + AST	Carbapenems
Gato et al. (2023)	Not stated (rapid)	MicroScan/VITEK, 48–72 h	Significant	Automated microdilution	Carbapenems (meropenem, ertapenem); carbapenemase types (OXA‐48, KPC)
Zhang et al. (2023)	Not stated	Disk/broth, ≥ 3–4 days	At least 1 day	Disk/broth	Carbapenems (ertapenem, imipenem, doripenem, meropenem)
Zeng et al. (2023)	Not stated	MIC/VITEK‐2	Not stated	MIC/VITEK‐2	Imipenem
Iskender et al. (2023)	Not stated (“rapid”)	Broth microdilution, days	Not stated	Broth microdilution	Colistin
Lin et al. (2024)	~3 days	Culture + ID + AST, ≥ 4 days	~1 day	Culture + ID + AST	Ceftazidime‐avibactam
Xu (2024)	~1 h (citing Yu et al.)	Not stated	Shortens reporting time	VITEK‐2, Carba NP	Carbapenems (ertapenem, imipenem, meropenem)
Jian et al. (2024b)	Minutes	VITEK‐2, 2–4 days	~1 day	Automated AST	Carbapenems (doripenem, imipenem), colistin
Jian et al. (2024a)	~3 days	VITEK‐2, up to 5 days	2 days	Automated AST	Levofloxacin, ciprofloxacin
Lopez‐Cortez et al. (2024)	Not stated (“rapid”)	AST, up to 72 h	Significant	AST	Ciprofloxacin, ceftriaxone, cefepime, meropenem, tobramycin
Xu and Gao (2025)	1–2 h	VITEK‐2 Compact, 1–2 days	1–2 days	Automated AST	Multiple antibiotics (piperacillin/tazobactam, ceftazidime, ceftriaxone, cefotetan, aztreonam, imipenem, amikacin, levofloxacin, Co‐trimoxazole)
Lin et al. (2025)	~3 days	VITEK‐2, 2–4 days	~1 day	Automated AST	Multiple antibiotics (12 antibiotics, including amikacin, gentamicin, piperacillin/tazobactam, ceftazidime, ceftriaxone, cefoperazone/sulbactam, flomoxef, cefepime, imipenem, doripenem, ciprofloxacin, levofloxacin)
Xu et al. (2025)	0.5 h	Culture + AST, > 24 h longer	Up to 24 h	Culture + AST	Carbapenems (ertapenem, imipenem, meropenem)
Ye et al. (2025)	20 min	PCR/gene sequencing (not specified)	Not stated	PCR/gene sequencing	Carbapenems; carbapenemase subtypes (KPC, NDM, OXA‐48)
López‐Cortés et al. (2025)	Not stated	Disk diffusion, 24–72 h	Not stated	Disk diffusion	Ciprofloxacin

Abbreviations: AST, aspartate aminotransferase; ID, identification; KPC, Klebsiella pneumoniae carbapenemase; MALDI, matrix‐assisted laser desorption/ionization; MIC, minimum inhibitiry concentration; ML, machine learning; MS, mass spectrometry; NDM, New Delhi metallo‐β‐lactamase; NP, Nitrocefin‐based Phenotypic Carbapenemase test; PCR, polymerase chain reaction; TOF, time‐of‐flight.

By contrast, traditional workflows most frequently used across the same studies included VITEK‐2 or related automated systems (seven studies), disk diffusion or E‐tests (four studies), and broth microdilution (two studies), alongside other phenotypic or molecular methods, such as polymerase chain reaction. These approaches generally required 24 h–5 days, depending on incubation and ID procedures. Even among rapid automated systems, such as VITEK‐2 Compact, completion commonly extended beyond a full day.

**Figure 4:**
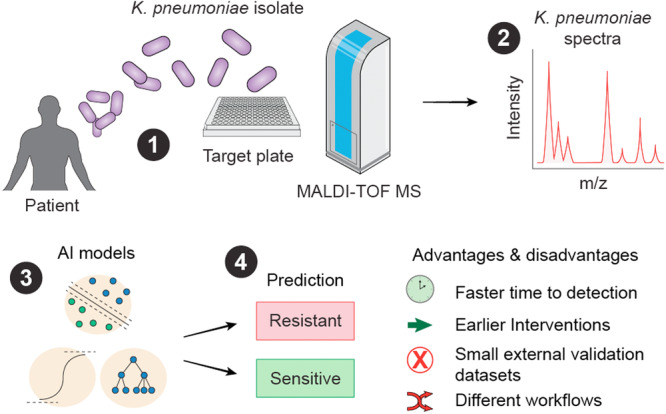
Artificial clinical decision support system workflow for *Klebsiella pneumoniae* resistance reporting. (1) A small amount of a bacterial colony is mixed with a matrix (commonly α‐cyano‐4‐hydroxycinnamic acid—CHCA), obtained from a patient. A pulsed UV laser hits the matrix‐bacteria crystals. The matrix absorbs the laser pulse, desorbing the analytes into the gas phase, ionizing them, usually forming singly charged ions, +1. The ions are accelerated toward the detector by an electric field. (2) This results in a signal intensity versus ion flight time and is converted into an *m/z* spectrum. (3) ML models such as support vector machines, logistic regression, and Random Forest use features derived from the spectra to make AMR predictions in *K. pneumoniae* (4). ML–MALDI–TOF–MS models can lead to faster resistance reporting, leading to earlier interventions, but are currently limited by insufficient external data sets to test the performance of models on unseen data, and inter‐laboratory workflows such as the use of different MALDI–TOF–MS instruments to obtain MALDI–TOF–MS spectra. Figure created using Adobe Illustrator version 30.0. AI, artificial intelligence; AMR, antimicrobial resistance; MALDI, matrix‐assisted laser desorption/ionization; ML, machine learning; MS, mass spectrometry; TOF, time‐of‐flight; UV, ultraviolet.

Quantitatively, the time savings attributable to ML‐based MALDI‐TOF ranged from ~ 18 h to more than 2 days, with individual reports documenting: 24–48 h saved for β‐lactamase detection (De Carolis et al. 2017), ~18.5 h saved in colistin testing (Giordano and Barnini 2018), up to 23 h saved for carbapenemase ID (Cordovana et al. 2018), ~46 h saved in ciprofloxacin resistance prediction (Wang et al. 2022), and 35–71 h saved when predicting carbapenem resistance directly from blood cultures (Yu et al. 2023). Collectively, these results are summarized in Table 3. Importantly, no study demonstrated a delay relative to the reference method, confirming that MALDI–TOF‐based ML frameworks either match or markedly exceed conventional diagnostics in speed, a key advantage of this approach (Figure 4). These collective findings illustrate that ML‐enhanced MALDI–TOF–MS transforms resistance detection from a multiday, culture‐dependent process into a workflow with improved time‐to‐resistance detection. This acceleration is a key factor for enabling earlier targeted therapy, optimizing patient outcomes, and supporting antimicrobial stewardship (Figure 4).

## 4 Discussion

Most of the evaluated ML models (*n* = 15/23) were developed to predict carbapenem resistance in *K. pneumoniae*, reflecting its critical clinical and epidemiological importance. According to the GLASS 2025 report, carbapenem resistance in *K. pneumoniae* bloodstream infections reached 41.2% in the South‐East Asia Region, with a global prevalence of 16.7% (95% CrI, 13.8–20.1) and a median annual relative increase of 15.3% between 2018 and 2023 (GLASS 2025). GLASS also reported that CRKP infections are associated with fatality rates exceeding 30%, highlighting them as major global health threats with limited therapeutic options (GLASS 2025). These findings justify the focus of MALDI–TOF–MS‐based ML research on carbapenem resistance. Rapid detection of this phenotype could substantially improve empirical therapy decisions and support antimicrobial stewardship.

Across the evaluated studies, only 8.7% (*n* = 2/23) assessed the impact of culture medium on the performance of ML models for antibiotic resistance prediction in *K. pneumoniae*. The apparent contradiction between Gato et al. (2023), which found significant media effects on ML model performance, and Cordovana et al. (2018), which found no differences, can be reconciled through several methodological distinctions. Gato et al. (2023) employed full‐spectrum RF analysis using all peaks as input features, while Cordovana et al. (2018) focused on automated detection of a single specific peak at 11,109 m/z associated with the pKpQIL plasmid. Full‐spectrum ML approaches that leverage the complete proteome for classification may appear more sensitive to media‐induced spectral variations than single‐biomarker detection methods.

Gato et al. (2023) attributed the media effect to biological differences in bacterial growth, influenced by different nutrients, which alter the protein expression profile captured by MALDI–TOF–MS. When classification relies on pattern‐matching across thousands of spectral features, even subtle changes in protein expression due to media composition may accumulate to affect prediction accuracy. For ML models utilizing full‐spectrum analysis, the predominant approach in recent studies, the evidence suggests that training and test isolates should be cultured on identical media to maximize prediction accuracy. The high performance achieved by studies using blood agar protocols (AUROC 0.95–1.00, Table S1) compared with the variable performance in studies with unreported conditions supports the recommendation for a standardized approach. To this end, Gato et al. (2021) developed a standardized operating procedure specifically designed to reduce variability and support interlaboratory reproducibility. Using blood agar exclusively, the RF algorithm achieved 100% accuracy for CPK ID with TIC normalization and selection of all peaks as input features. Here, the authors note, spectral variability could be reduced by developing standardized protocols.

The time savings achieved by MALDI–TOF–MS‐ML models are clinically significant, particularly in the context of CRKP outbreaks where delayed detection contributes toward transmission. ML‐assisted MALDI‐TOF workflows consistently reduced diagnostic turnaround by 18 h to more than 2 days compared with standard automated or phenotypic methods. Yu et al. (2023) achieved 35–71 h faster CRKP reporting from blood cultures, while De Carolis et al. (2017) and Cordovana et al. (2018) demonstrated savings of 24–48 h and up to 23 h, respectively. In a recently reported CRKP outbreak, diagnostic delays and inadequate early isolation contributed to a nosocomial infection rate of 8.1% and multiple fatalities (Pang et al. 2025). Earlier recognition of resistance, within hours rather than days, could have allowed rapid cohorting of infected patients and timely optimization of therapy, potentially averting onward spread. Integrating MALDI–TOF–MS‐ML pipelines into ICU workflows could help manage such outbreaks, transforming resistance detection from a retrospective confirmation step into a proactive infection‐control measure.

Across the evaluated studies, RF emerged as the most frequently developed and best‐performing ML model for predicting AMR using MALDI–TOF–MS spectra. In this evaluation, 13 of 23 studies employed RF algorithms, with several identifying it as the top classifier for distinguishing resistant from susceptible *K. pneumoniae* isolates, particularly for carbapenem resistance, where reported AUROC values commonly exceeded 0.95. This strong performance can be attributed to the RF model's ensemble structure, which combines multiple decorrelated decision trees to handle the high‐dimensional, nonlinear, and noise‐prone nature of proteomic spectral data. Recently, an RF‐based workflow applied to over 4000 MALDI–TOF–MS spectra of *Staphylococcus epidermidis* achieved AUROC scores up to 0.95 and AUPRC up to 0.97, outperforming LR and naïve Bayes models (Ren et al. 2024). These results reinforce that ensemble methods, such as RF, are particularly well‐suited to MALDI–TOF–MS data, offering robust, generalizable performance for rapid and accurate prediction of resistance phenotypes in *K. pneumoniae*.

Only one study directly validated normalization effectiveness, demonstrating that TIC normalization reduced interlaboratory coefficient of variation by 40% compared with raw spectra (Gato et al. 2021). Despite methodological heterogeneity, high‐performing ML models (AUROC > 0.9 (Table S1) were achieved across diverse normalization strategies, indicating that model architecture and feature selection may partially compensate for preprocessing variations. However, the absence of systematic head‐to‐head comparisons across studies precludes definitive conclusions regarding optimal normalization approaches, and the need for standardization in spectrum acquisition and preprocessing has been identified.

A key limitation identified across the evaluated studies is the limited scope of external validation data sets, which constrains the generalizability of the ML models developed for predicting antibiotic resistance in *K. pneumoniae*. While internal train‐test splits were consistently applied, only 3, 13.0% (*n* = 3/23) of the studies performed independent external validation. This imbalance increases the risk of overfitting, as models may learn data set‐specific noise, such as spectral artefacts, instrument calibration patterns, or strain distributions unique to the training site, rather than genuine biological markers of resistance. When evaluated on small or homogeneous data sets, these models are therefore insufficiently challenged to demonstrate true generalizability, leading to a potential overestimation of performance. This problem is particularly relevant to MALDI–TOF–MS data, which are inherently high‐dimensional and susceptible to inter‐laboratory variability. Consequently, although ensemble algorithms such as RF have shown excellent internal performance, their clinical utility remains limited until larger, multicenter validation data sets are used to ensure robustness across different instruments, laboratories, and epidemiological settings.

Additionally, independent external validation data sets may be difficult to obtain because patient privacy regulations and institutional governance policies restrict cross‐site data sharing (Jannace et al. 2024). Further, substantial heterogeneity in MALDI–TOF–MS platforms, acquisition parameters, and preprocessing pipelines reduces interoperability between data sets. Together, these factors make it challenging to assemble sufficiently large, multicenter cohorts for robust external validation.

Among the 23 studies evaluated, most used either the Bruker Microflex LT/SH or the bioMérieux VITEK MS systems, reflecting their dominance in clinical microbiology but also revealing a source of data heterogeneity. Bruker instruments generate high‐resolution, continuous full spectra, while VITEK MS can produce preprocessed, peak‐limited profiles of around 200 discrete *m/z* values. This difference significantly affects the comparability of spectral data. These discrepancies limit model generalizability and undermine the effectiveness of transfer learning. López‐Cortés et al. (2025) demonstrated that a CatBoost model trained on Bruker full‐spectrum data achieved an AUROC of 0.73 for predicting ciprofloxacin resistance in *K. pneumoniae*, whereas the same pretrained model transferred to VITEK MS data performed poorly (AUROC = 0.58; AUPRC = 0.71). Attempts to reconcile these differences through spectral alignment and zero‐padding missing features did not restore lost information. The lack of standardized mass spectral data formats and preprocessing pipelines hampers the cross‐platform transferability of ML models, thereby restricting their practical deployment in diverse clinical laboratories.

Studies using bioMérieux instruments showed greater variability in reported normalization methods, with several not reporting methods at all, potentially reflecting reliance on instrument‐integrated preprocessing (Huang et al. 2020; Xu 2024; Xu et al. 2025; Xu and Gao 2025). In contrast, studies using Bruker platforms more frequently employed third‐party software (MaldiQuant, Clover MS) with explicitly documented normalization methods (Gato et al. 2021; Weis et al. 2022; Yu et al. 2023).

Despite this, the integration of MALDI–TOF–MS with ML represents an advance in the rapid prediction of AMR in *K. pneumoniae*, offering substantial diagnostic time savings and high resistance determination potential, though larger, multicenter validation studies remain essential to ensure clinical robustness and widespread implementation.

## Author Contributions

**Stephen Mark Edward Fordham:** conceptualization, investigation, writing – original draft, writing – review and editing, visualization, methodology, formal analysis, data curation, project administration.

## Funding

The author received no specific funding for this work.

## Ethics Statement

The author has nothing to report.

## Conflicts of Interest

None declared.

## Supporting information


**Supplementary Table S1:** Data extraction from MALDI–TOF–MS studies using machine learning models for antibiotic resistance prediction in *Klebsiella pneumoniae*.

## Data Availability

Data that support the findings of this study are available in the Supporting Information of this article.
